# Biocompatibility and Cell Death Mechanisms Induced by PMMA-Based Dental Materials in Gingival Fibroblasts and OECM-1 Tumor Cells

**DOI:** 10.3390/dj14050315

**Published:** 2026-05-21

**Authors:** Florentina Rus, Radu Radulescu, Alexandra Popa, Bianca Voicu-Balasea, Monica Musteanu, Melis Izet, Corina Muscurel, Lucian Toma Ciocan, Sebastian-Andrei Bancu, Ana Cernega, Alexandra Ripszky, Silviu-Mirel Pituru

**Affiliations:** 1 Department of Biochemistry, Faculty of Dental Medicine, University of Medicine and Pharmacy Carol Davila, 8 Eroilor Sanitari Blvd, 050474 Bucharest, Romania; florentina.rus-hrincu@umfcd.ro (F.R.); radu.radulescu@umfcd.ro (R.R.); sebastian-andrei.bancu@drd.umfcd.ro (S.-A.B.); alexandra.ripszky@umfcd.ro (A.R.); 2 The Interdisciplinary Center for Dental Research and Development, Faculty of Dental Medicine, University of Medicine and Pharmacy Carol Davila, 17-23 Plevnei Street, 020021 Bucharest, Romania; melis.izet@yahoo.com; 3 Department of Biochemistry and Molecular Biology, Faculty of Pharmacy, University Complutense of Madrid, 28040 Madrid, Spain; mmustean@ucm.es; 4 Department of Biochemistry, Faculty of Medicine, University of Medicine and Pharmacy Carol Davila, 8 Eroilor Sanitari Blvd, 050474 Bucharest, Romania; corina.muscurel@umfcd.ro; 5 Department of Complete Denture, Faculty of Dental Medicine, University of Medicine and Pharmacy Carol Davila, 17-23 Plevnei Street, 010221 Bucharest, Romania; lucian.ciocan@umfcd.ro; 6 Department of Organization, Professional Legislation and Management of the Dental Office, Faculty of Dental Medicine, University of Medicine and Pharmacy Carol Davila, 17-23 Plevnei Street, 020021 Bucharest, Romania; ana.cernega@umfcd.ro (A.C.); silviu.pituru@umfcd.ro (S.-M.P.)

**Keywords:** 3D printing, PMMA, human oral squamous carcinoma cell line, biocompatibility, autophagy, apoptosis, human gingival fibroblasts, provisional prosthetic restauration

## Abstract

**Background/Objectives:** The present study aims to test three different types of PMMA (Fotodent Guide—3D printed (M1), Aidite Temp—milled (M2), Duracryl—self-polymerized (M3) on HFIB-G and on OECM-1. **Methods:** The two cell types (HFIB-G and OECM-1) were kept in contact with the materials, Fotodent Guide, Aidite Temp, and Duracryl (*n* = 6), for 24 and 48 h, and subsequently subjected to the following tests: MTT, LDH, NO (according to ISO 10993-5:2009), and immunofluorescent detection of proteins associated with autophagy and apoptosis (mitochondria and caspases 3/7; detection of autophagosomes). Statistical interpretation was made using t-test and ANOVA (* *p* < 0.05; ** *p* < 0.01; *** *p* < 0.001). **Results:** The MTT assay revealed a reduction in cell viability for all tested materials on gingival fibroblasts compared to control cells, with the most pronounced decrease observed for the 3D-printed material (M1 viability 66.77% for 24 and 52.45% 48 h—*p* < 0.001), while the self-polymerizing resin (M3 viability 85.92% for 24 h and 85.63% for 48 h) showed the highest level of cellular tolerance (*p* < 0.001 at 24 h and *p* < 0.01 at 48 h). Regarding OECM-1 cells, all materials reduced cell viability, particularly M3 after 48 h of incubation (viability 61.79%—*p* < 0.001). LDH levels generally indicated low membrane damage for all materials. Statistically significant increases in NO levels were recorded for both cell types, suggesting a mild proinflammatory response, especially for M2 OECM-1 48 h—*p* < 0.05 and M3 (HFIB-G 48 h—*p* < 0.05, OECM-1 48 h *p* < 0.05). For both 24 and 48 h, fluorescence analysis demonstrated a significant increase in mitochondrial activity in gingival fibroblasts (*p* < 0.001), whereas tumor cells exhibited a significantly decreased mitochondrial activity (*p* < 0.001), particularly for the 3D-printed material M1 (*p* < 0.001). Caspase-3/7 expression increased in gingival fibroblasts incubated with materials for 24 and 48 h (*p* < 0.001), while tumor cells showed reduced caspase activity both after 24 and 48 h (*p* < 0.001). Autophagosome formation decreased initially in fibroblasts at 24 h (*p* < 0.001) but increased significantly after 48 h (*p* < 0.001), while tumor cells generally showed enhanced autophagic activity under most experimental conditions (*p* < 0.001). **Conclusions:** Our results suggest that all three PMMA-based materials exhibit acceptable biocompatibility profiles, of more than 70%, according to ISO 10993-5:2009, although cellular responses vary depending on the manufacturing technique and the cellular model used. In our study conditions, self-polymerized resin (M3) was the most compatible with gingival fibroblasts, while the 3D-printed and CAD/CAM milled materials (M1 and M2) had a more pronounced impact on cells’ viability and metabolic activity.

## 1. Introduction

Provisional restorations play a crucial stage in oral rehabilitation, having a protective role both for prepared teeth (prosthetic abutments) and for the adjacent soft tissues until the application of definitive restoration [1]. These types of restorations ensure the maintenance of oral functions during the interval between preparation and cementation of the final work. In addition, they also provide diagnostic and therapeutic functions, allowing the evaluation and optimization of functional (dynamic and static occlusion relationships, vertical occlusion dimension) and biological (gingival healing and remodeling) parameters [1,2,3].

Although these types of restorations are usually worn for a short period, there are also cases of complex oral rehabilitations, which involve multidisciplinary approaches (surgical, periodontal, orthodontic treatments, etc.), during which the wearing period of the provisional restorations can be significantly extended [1,4,5].

Given the prolonged contact of temporary restorations with oral tissues, the selection of material becomes a key factor. They must be manufactured from a material with a favorable biological profile to reduce the incidence of adverse reactions at the level of periodontal or peri-implant tissues [6,7].

Over time, a wide range of materials have been proposed and used for the manufacture of temporary prosthetic parts (wood, porcelain, etc.), but polymethylmethacrylate (PMMA) has emerged as the reference material. It is based on methacrylate monomers, which, through polymerization reactions, form a solid and relatively stable polymer matrix. PMMA is one of the most widely used polymeric materials in dentistry, especially for temporary prosthetic restorations. Resin-based materials can release various components into the oral environment, which can exert harmful biological effects on gingival tissue [8].

Although the cost is relatively low, the esthetics are satisfactory, and the processing is easy, the clinical performance of PMMA is limited by a series of structural (increased porosity, low mechanical strength, polymerization shrinkage, chromatic instability over time) and biological characteristics (release of residual methacrylate monomer, considered cytotoxic—potentially allergenic and inflammatory) [9]. Resin-based materials may release various components into the oral environment, which can potentially exert harmful biological effects on gingival healthy tissues and probably on the tumoral tissues also [9,10]. A major concern is incomplete polymerization resins, with studies reporting monomer-to-polymer conversion rates of 60–75% [11,12]. Additionally, exposure to oxygen inhibits polymerization at the surface layers, and so, subsequent polishing procedures may lead to the release of residual monomers [13,14,15]. These limitations have led to the optimization of material composition and the emergence of new processing techniques [16,17].

In this context, digital CAD/CAM technologies, both additive (3D-printed resins) and subtractive (milled PMMA), have been integrated into the manufacturing process of temporary dentures. The manufacturing method of the prostheses could significantly influence the amount of residual monomer present in PMMA-based resins, and by extension, their potential to be released into the oral cavity—a factor correlated with allergic and cytotoxic reactions [18,19,20]. Smidt et al. showed in their study that no residual monomer was identified in organic solutions from 3D-printed materials, while milled and conventional/self-polymerizing resins released significant amounts of residual monomer [21].

Methacrylate-based dental resins can be enhanced through the incorporation of various modifiers, leading to improved mechanical performance, reduced polymerization shrinkage, and increased durability, albeit with potential trade-offs in conversion efficiency and water sorption. Polymethyl methacrylate exhibits favorable biocompatibility and low shrinkage stress but is limited by inferior mechanical strength and wear resistance compared to highly crosslinked systems such as Bis-GMA-based resins [22].

Aryloxycyclophosphazene-modified methacrylate systems, derived from Cyclophosphazene structures, demonstrate potential for enhanced toughness, thermal stability, and reduced internal stress through multifunctional crosslinking mechanisms. Overall, while conventional dimethacrylate resins remain the clinical standard, phosphazene-modified materials represent a promising direction for the development of advanced restorative composites with optimized mechanical and physicochemical properties [22].

Regardless of the technology used, conventional, additive, or subtractive, materials intended for temporary restorations go through polymerization stages during the manufacturing process. Thus, the parameters of this process directly influence the degree of monomer conversion. Prolonged polymerization cycles and high temperatures lead to a significant reduction in the number of unreacted monomers, but not to their complete elimination [5,12,23,24]. Although some of these residual monomers may remain entrapped in the polymer matrix after polymerization, over time, under the action of mechanical factors and the interaction with oral bacterial enzymes and saliva, PMMAs may undergo progressive degradation, thus favoring the release of residual monomers [25,26,27].

Exploring the mechanism of action of resin monomers offers an important opportunity to gain valuable insights for the development of improved therapeutic approaches in dental restorative biomaterial and protecting oral tissues. Current evidence indicates that resin monomers can induce intracellular redox imbalance through increased production of reactive oxygen species (ROS), thereby modulating multiple interconnected signaling pathways involved in apoptosis and cell survive [28]. Nevertheless, the precise intracellular mechanism and specific signaling pathways underlying resin monomer-induced redox imbalance remain incompletely understood [29,30,31].

Autophagy is a highly conserved and tightly regulated cellular process present in all cell types, contributing to both cell survival and cell death [32]. This role depends on the cellular context and the environmental stress conditions [32]. By degrading damaged organelles and proteins, this process helps maintain cellular homeostasis and supports cell survival. Various forms of cytotoxic stress, including exposure to toxins and oxidative stress, can activate autophagy as a defensive response for restoring cellular integrity. However, if the autophagic response is inadequate, cell death may occur. Autophagosomes are key structures in this process, representing the central components of the degradation and recycling system of cellular components, which is the reason why monitoring autophagosome formation is useful to obtain important insights into autophagy progression [32,33]. Therefore, to evaluate the intensity of autophagic activity in our study, we determined the fluorescent detection of autophagosomes.

Apoptosis is a tightly regulated and programmed form of cell death. This is essential for normal development and maintaining tissue homeostasis. Elevated levels of cytotoxic stress can trigger apoptosis through the activation of specific signaling pathways. The extension of cytotoxicity determines whether a cell undergoes apoptosis or remain viable [34].

Autophagy and apoptosis are closely interconnected processes that influence cell fate [34]. Autophagy generally promotes cell survival by degrading damaged organelles and different proteins that could otherwise initiate cell death. However, excessive autophagic activity may lead to autophagic cell death, a distinct process from apoptosis [35]. In many cases, autophagy precedes apoptosis by depleting survival factors and facilitating the activation of apoptotic signaling pathways [35].

Taking into consideration that caspases 3 and 7 are commonly activated during the late stages of apoptosis, and their activation serves as a hallmark of programmed cell death, in the present study, apoptosis was assessed using fluorescent detection of caspase-3/7 activity.

Mitochondrial activity is crucial in cells exposed to various materials (nanomaterials, pollutants, drugs, biomaterials) because mitochondria are the main subcellular targets of toxicity and key regulators of the stress response. These organelles not only produce the energy (ATP) needed to manage the impact of materials but also undergo structural and functional changes that determine cell fate (survival or death) [36]. Mitochondrial damage triggered by the materials leads to the release of cytochrome C, a crucial step in the apoptosis pathway, leading to cell death [37]. Mitochondrial activity in cells exposed to materials is a barometer of cellular health, dictating how the cell responds to toxicity, manages oxidative stress, and ultimately whether it will survive or undergo apoptosis [36].

Considering these aspects, a comparative evaluation of the different types of PMMA, depending on the processing method, is required to assess how they correlate with the molecular response and the amount of residual monomer released. Identifying the manufacturing method that ensures a low cytotoxic response and a favorable biological profile is essential, as it can be correlated with the lowest amount of residual monomer released [16,38].

Our study aims to test three different types of PMMA (Fotodent Guide—3D printed, Aidite Temp—milled, Duracryl—self-polymerized) both on normal human gingival fibroblasts, to allow the evaluation of biocompatibility under normal conditions, representative of healthy oral tissues, and on tumoral cells, to follow the analysis of the cellular response in a pathological context, which is relevant for patients with a history of oral oncology undergoing prosthetic rehabilitation. This comparative study provides an overview of the biological safety of PMMA-based materials, in accordance with ISO 10993-5:2009 [39], reflecting clinical situations in which they may come into contact with both healthy tissues and neoplastic tests. Testing the biocompatibility of PMMA-based dental materials at 24 and 48 h is essential to assess both acute cytotoxic effects and their temporal evolution, enabling differentiation between transient and persistent cellular responses [39].

## 2. Materials and Methods

This study was designed as an in vitro controlled experimental study using a 3 × 2 factorial design to evaluate the biological response of two cell lines (HFIB and OECM) to three types of PMMA-based dental materials (3D-printed, milled, and autopolymerizable).

### 2.1. Sample Preparation

#### 2.1.1. Samples Material Composition

The development of dental materials for provisional prosthetic restorations focuses on improving mechanical properties, durability, and biocompatibility, and reducing polymerization shrinkage through filler innovation and binder modification [22]. The incorporation of advanced fillers and the chemical modification of resin matrices, including systems based on Bis-GMA, enhance strength, adhesion, and mineralization potential while maintaining antibacterial performance. Overall, the design of modified methacrylate-based systems with improved crosslinking and filler–matrix interactions represents a key strategy for achieving superior mechanical performance in provisional dental restorations [22].

In the present study, three types of resin-based composite dental materials were analyzed, obtained through different fabrication techniques: M1 (FotoDent Guide; Dreve Dentamid GmbH, Unna, Germany), manufactured by 3D printing; M2 (Aidite Temp, Aidite (Qinhuangdao)Technology Co., Ltd., Qinhuangdao, China), produced by CAD/CAM milling Planmeca PlanMill 60 S unit (PLANMECA, Helsinki, Finland); and M3 (Duracryl, New stetic, Guarne (Antioquia), Colombia), obtained through self-polymerization. Chemical composition of the materials used in the current study is detailed in Table 1.

**Table 1:** Chemical composition of dental material used [40,41,42].

Nr. Crt.	Dental Material Name	Material Type	Chemical Composition (According to Supplier Insert)
M1	Fotodent guide (PMMA)	3D-printable light-curing resin (drill guide resin)	Methacrylate-based mixture; hazardous ingredients listed include: - 7,7,9(7,9,9)-trimethyl-4, 13-dioxo-3, 14-dioxa-5, 12-diazahexadecane-1,16-diylbismethacrylate < 10%. - 2-hydroxyethyl methacrylate < 6.3%. - Aliphatic urethane methacrylate < 10%. - Diphenyl (2,4,6-trimethylbenzoyl) phosphine oxide < 10%. - Hydroxypropyl methacrylate < 10%. - Acrylic resin < 3.6%. Further ingredients: - Bumetrizole < 1%.
M2	Aidite Temp (PMMA)	CAD/CAM-milled PMMA (pre-polymerized disk/block)	99% polymethylmethacrylate (PMMA)
M3	Duracryl (PMMA)	Self-curing/auto-polymerizing acrylic resin (powder–liquid system)	Polymer: ethyl and methyl methacrylate copolymer (polymer component). Monomer components listed include methyl methacrylate (MMA), ethylene glycol amine-type chemical initiator (plus pigments/additives).

#### 2.1.2. Digital Processing of the Composite Materials Under Investigation

The composite materials included in the present study were processed using two digital fabrication techniques:

- The subtractive method (CAD/CAM);
- The additive method (3D printing)

Subtractive Method (CAD/CAM)

The specimens were fabricated by milling industrially prefabricated Voco Grandio disks using a Planmeca PlanMill 60 S unit (PLANMECA, Helsinki, Finland). The Planmeca PlanMill 60 S is a 5-axis dental milling system designed for both laboratory and chairside applications. In combination with CAD/CAM software PlanCAM version 3.1, it enables the precise fabrication of dental restorations, implant abutments, and various prosthetic components from materials such as zirconia, lithium disilicate, PMMA, and wax. The system supports both wet and dry milling and is equipped with an automatic tool changer to enhance operational efficiency [9,18].

Additive Method (3D Printing)

Discoidal specimens were produced using a digital light processing (DLP) technique with the Anycubic Mono X 6Ks printer (Hongkong Anycubic Technology Co., Hongkong, China). This approach allowed the fabrication of samples with well-defined morphological and structural characteristics, required for subsequent analyses.

The Anycubic Mono X 6Ks (Hongkong Anycubic Technology Co., Hongkong, China) is a monochrome LCD-based mSLA printer operating at a wavelength of 405 nm. It provides a 6K resolution (5760 × 3600 pixels) on a 9.1-inch screen, with an effective pixel size of 34.4 µm. The layer thickness is adjustable, starting from 10 µm. The printer operates within an open system, compatible with proprietary software (Anycubic Photon Workshop 3.0) as well as third-party slicing software [9].

From the investigated materials, discoidal specimens with a diameter of 10 mm and a thickness of 2 mm were fabricated according to the manufacturers’ protocols [40,41,42].

### 2.2. Cell Culture

Two cell models were used in this study: human gingival fibroblast cell line (HFIB-G Provitro, Berlin, Germany, Cat. No. 121 0412; It is not registered in any international database.Catalog number: 121 0412) and the human oral squamous carcinoma cell line (OECM-1 Sigma-Aldrich, Darmstadt, Germany, Cat. No. SCC180; RRID: CVCL_6782; Data base name: Cellosaurus; Accesion number: CVCL_6782).

HFIB-G cells were cultured in Dulbecco’s Modified Eagle Medium (DMEM) supplemented with 10% fetal bovine serum (FBS) and 1% antibiotic–antimycotic solution, while OECM-1 cells were maintained in RPMI 1640 medium supplemented with 5% FBS and 1% penicillin/streptomycin/amphotericin. Both cell lines were incubated at 37 °C in a humidified atmosphere containing 5% CO_2_ [9].

Cells were seeded in 24-well plates at a density of 2 × 10^4^ cells per well and allowed to adhere overnight. Subsequently, the cultures were exposed to sterilized dental material specimens for 24 or 48 h, depending on the specific assay performed. All biological assays (MTT, LDH, NO, apoptosis, and autophagy) were performed at both time points. After the incubation period, the material specimens were removed using sterile forceps. Control groups consisted of cells cultured in the absence of dental materials.

Prior to cell exposure, all material specimens were sterilized by immersion in alcohol for 5 min, followed by ultraviolet (UV) irradiation for 30 min on each side [9,24,28,39].

A 70% ethanol treatment followed by ultraviolet (UV) exposure is widely regarded as an optimal sterilization approach for PMMA-based dental materials in in vitro studies, as it effectively reduces microbial contamination while preserving the material’s integrity. This method avoids the high temperatures or reactive conditions associated with other sterilization techniques that may alter the physicochemical properties of the polymer. Importantly, the combined ethanol–UV protocol does not significantly affect the composition, surface characteristics, or residual monomer content of the tested materials, thereby minimizing the risk of introducing experimental artifacts and ensuring the reliability of subsequent biocompatibility assessments [24,28,39].

The tested materials were fabricated by self-polymerization, 3D printing, and CAD/CAM milling [43].

### 2.3. Cell Viability Assay (MTT Assay)

Cell viability was evaluated after 24 and 48 h of exposure to the tested dental material specimens in HFIB-G and OECM-1 cells using the MTT assay. A 1 mg/mL solution of 3-(4,5-dimethylthiazol-2-yl)-2,5-diphenyltetrazolium bromide (MTT) (Sigma-Aldrich, Darmstadt, Germany) was used to assess metabolic activity.

Following the incubation period with the materials, cells were incubated with the MTT solution for 3 h at 37 °C. MTT, a yellow tetrazolium salt, is reduced by metabolically active cells to insoluble purple formazan crystals. The resulting formazan crystals were dissolved in isopropanol, and absorbance was measured at 595 nm using a FLUOstar^®^ Omega multimode microplate reader (BMG LABTECH, Ortenberg, Germany) [9].

The intensity of the purple coloration is directly proportional to the number of viable cells [43,44].

### 2.4. Level of Lactate Dehydrogenase (LDH Assay)

The extracellular release of the cytoplasmic enzyme lactate dehydrogenase (LDH) serves as an indicator of membrane integrity loss and, consequently, of cellular damage or cell death. The assay is based on the enzymatic conversion of lactate to pyruvate catalyzed by LDH, accompanied by the reduction in NAD^+^ to NADH.

To assess cytotoxicity, LDH levels were quantified in samples previously incubated for 24 and 48 h with HFIB-G cells and OECM-1 cells using a commercial LDH Cytotoxicity Assay kit (Thermo Fisher Scientific, Eugene, OR, USA).

A volume of 50 μL from each sample was mixed with 50 μL of the specific reaction solution. After a 30 min incubation period, absorbance was measured at 490 nm and 680 nm using a FLUOstar^®^ Omega multimode microplate reader. The absorbance value recorded at 680 nm was subtracted from that obtained at 490 nm to correct for background interference [9,44].

### 2.5. Level of Nitric Oxide (Griess Assay)

Nitric oxide production was indirectly assessed by quantifying nitrite levels using the Griess reaction. This method is based on the interaction between nitrite and Griess reagents, leading to the formation of a chromophoric pink compound that can be detected spectrophotometrically at 540 nm.

Nitric oxide levels were determined in the culture medium after 24 and 48 h of incubation of the samples with HFIB-G cells and OECM-1 cells, using a commercially available Nitric Oxide Assay Kit (Thermo Fisher Scientific, USA), following the manufacturer’s instructions.

The absorbance was recorded at 540 nm using a FLUOstar^®^ Omega multimode microplate reader (BMG LABTECH) [9].

### 2.6. Mitochondria and Caspase-3/7 Immunofluorescent Detection (Late Apoptosis)

Mitochondrial activity in HFIB-G cells and OECM-1 cells was evaluated using the BioTracker 633 Red Mitochondria Dye fluorescent kit (SCT137, Merck, Darmstadt, Germany), following the manufacturer’s protocol. This fluorogenic, membrane-permeable dye selectively stains mitochondria in viable cells. Loss of fluorescence signal occurs during cell death, as mitochondrial membrane depolarization leads to reduced dye retention.

After incubation with the tested samples, cells were stained and incubated for 15 min at 37 °C prior to analysis [9].

Late apoptosis was further assessed using the NucView 488 Caspase-3/7 Assay Kit for Live Cells (Biotium, Fremont, CA, USA, Cat. No. 30029-T), according to the manufacturer’s instructions. This assay enables the identification of apoptotic cells based on caspase-3/7 enzymatic activity through fluorescence microscopy. In apoptotic cells, activated caspase-3/7 cleaves the substrate, releasing a DNA-binding dye that translocates to the nucleus and emits bright green fluorescence upon binding to DNA.

Briefly, cells incubated for 24 and 48 h were stained, followed by a 30 min incubation period before analysis.

Fluorescently labeled cells were examined using an IM-3LD4D microscope (Optika, Ponteranica, Italy), and images were processed with ImageJ software, version 1.46j [9].

### 2.7. Autophagosome Immunofluorescent Detection

Autophagic activity in HFIB-G cells and OECM-1 cells following 24 and 48 h of incubation with the tested samples was evaluated using the Autophagy Assay Kit MAK138 (Sigma-Aldrich, Merck for HFIB-G cells) and Autophagy/Citotoxicity Dual Staining kit (Abcam, Danaher Co., Cambridge, UK for OECM-1 cells), according to the manufacturer’s instructions.

This assay enables the direct detection of autophagosomes in various cell types through a proprietary fluorescent marker specific for autophagic vesicles (λ_ex = 333 nm /λ_em = 518 nm).

After the incubation period, the culture medium was carefully removed, and the working solution of the autophagosome detection reagent was added to the cells. The cells were then incubated for 30 min at 37 °C prior to analysis.

Fluorescently stained cells were observed using an IM-3LD4D microscope (Optika, Italy), and the acquired images were processed using ImageJ software [9].

### 2.8. Statistical Interpretation

Our study results were statistically processed using Student’s *t*-test (Microsoft Office Excel) and expressed as mean value ± standard deviation (SD) (*n* = 6). A value of *p* less than 0.05 was considered statistically significant.

Additionally, the statistical analysis was performed using IBM SPSS Statistics, version 26. The distribution of quantitative variables was assessed using the Shapiro–Wilk test, and the homogeneity of variances was evaluated using the Levene’s test.

For parameters that met the assumptions of normal distribution and homogeneity of variances (LDH and MTT in HFIB and NO and MTT in OECM-1), differences between independent groups were analyzed using one-way analysis of variance (one-way ANOVA). When statistically significant differences were identified, post hoc comparisons were performed using the Tukey HSD test. For the NO parameter (in HFIB) and LDH (in OECM-1), for which the normality criterion was not met in all groups, intergroup differences were analyzed using the nonparametric Kruskal–Wallis H test. In cases where trends or differences close to the significance threshold were observed, pairwise comparisons were performed using the Mann–Whitney U test, with Bonferroni correction applied for multiple comparisons. The analyses were performed separately for each time point (24 h and 48 h). All statistical tests were two-tailed, and the threshold for statistical significance was set at α = 0.05. For the nonparametric post hoc analyses, the significance threshold was adjusted using the Bonferroni correction (α = 0.0167). For the fluorescence-analyzed parameters (the statistical analysis was performed using IBM SPSS Statistics, version 26). The distribution of quantitative variables (mitochondria and caspase-3/7 activity) was assessed using the Shapiro–Wilk test. As the data did not meet normality assumptions across all analyzed groups at both 24 h and 48 h, nonparametric statistical methods were employed. Quantitative variables were expressed as median and interquartile range. Differences between independent groups were analyzed using the Kruskal–Wallis H test. When statistically significant differences were identified, post hoc pairwise comparisons between groups were conducted using the Mann–Whitney U test, with Bonferroni correction applied for multiple comparisons. In autophagosome formation, at 24 h, the data exhibited a normal distribution in most groups; however, the test for homogeneity of variances (Levene’s test) indicated heterogeneity (*p* < 0.05). Under these conditions, differences between independent groups were analyzed using the nonparametric Kruskal–Wallis H test. When statistically significant differences were identified, post hoc comparisons were conducted using the Mann–Whitney U test, with Bonferroni correction applied for multiple comparisons. At 48 h, the data met the assumptions of normality and homogeneity of variances; therefore, comparisons between independent groups were performed using one-way analysis of variance (one-way ANOVA). When statistically significant results were obtained, post hoc comparisons were carried out using the Tukey HSD test. All statistical tests were two-tailed, and the threshold for statistical significance was set at α = 0.05.

The test results of the present study were reported to the values obtained for the control wells and represented graphically. The values used in constructing the graphs represent the arithmetic mean of the results obtained corresponding to each type of test in particular.

## 3. Results

### 3.1. Viability and Cytotoxicity Tests Results

**Figure 1:**
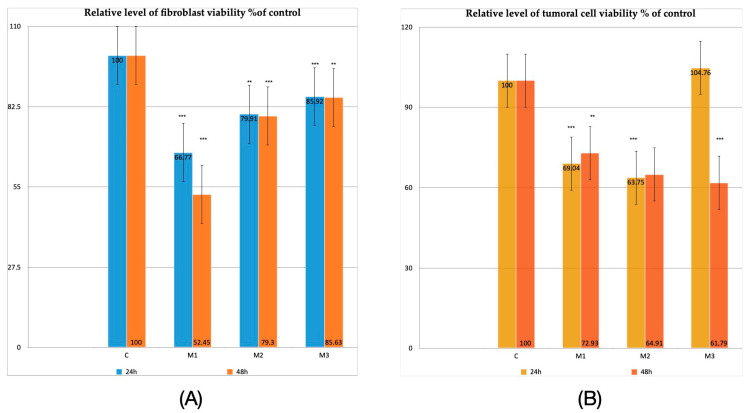
Viability results. (**A**) Relative level of fibroblasts viability % of control at 24 h and 48 h; (**B**) relative level of tumoral OECM-1 viability % of control at 24 h and 48 h. Results are means ± SD (*n* = 6) ** *p* < 0.01; *** *p* < 0.001.

The biocompatibility study was based on biological tests conducted according to ISO standards 10993-5:2009 (Biological evaluation of medical devices; Part 5: tests for in vitro cytotoxicity) [39]. Cell viability of gingival fibroblasts in M1 material decreased by 33.23% (*p* < 0.001) after 24 h and by 47.55% (*p* < 0.001) after 48 h. In M2, when incubating the material with gingival fibroblasts, a similar decrease was observed both at 24 h (*p* = 0.001) and at 48 h of approximately 20.5% (*p* < 0.001), while M3 incubated with gingival fibroblasts recorded the best cell viability, decreasing by approximately 16% (*p* < 0.001) both at 24 h and at 48 h (*p* = 0.012) (Figure 1A).

M1 samples incubated with OECM-1 tumoral cells after 24 h showed a decrease of 30.96% (*p* < 0.001). Similar results were obtained at 48 h— 27.07% (*p* = 0.002). Incubation of M2 with tumoral cells resulted in a more pronounced decrease—36.25% (*p* < 0.001) for 24 h and 35.09% (without statistical relevance) for 48 h. When incubating M3 with tumoral OECM-1 cells, a slight increase in cell viability of almost 5% (without statistical relevance) was observed at 24 h, followed by a dramatic decrease of 38.21% (*p* < 0.001) at 48 h (Figure 1B).

**Figure 2:**
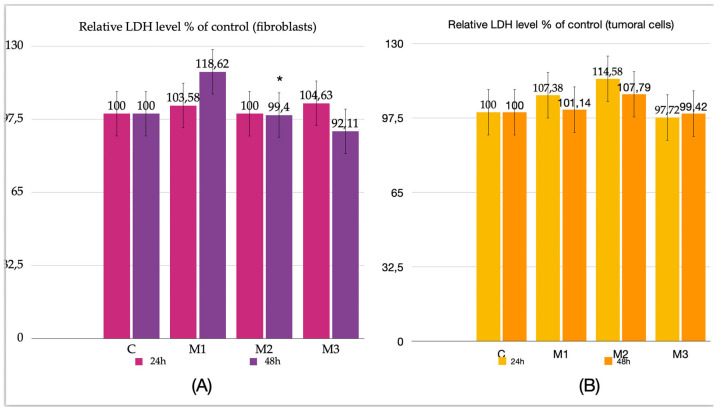
Cytotoxicity results, (**A**) Relative level of LDH % of control at 24 h and 48 h in fibroblasts; (**B**) relative level of LDH % of control at 24 h and 48 h in tumoral cells. Results are means ± SD (*n* = 6) * *p* < 0.05.

For all tested materials, at both the 24 h and 48 h time points, the LDH assay revealed only minor variations that did not reach statistical significance, with the exception of sample M2 at 48 h, where a statistically significant decrease was observed (*p* = 0.016) (Figure 2A).

In the case of material incubation with tumor cells, no statistically significant changes were detected at either 24 h or 48 h. These findings suggest that the tested materials do not exert a significant cytotoxic effect, as the observed increases were not statistically significant (Figure 2B).

The results obtained by the Griess test did not reveal significant differences—only moderate increases were observed, ranging from approximately 3% to 8.69%. The maximum value was recorded in the case of gingival fibroblasts incubated for 48 h hours in the presence of M3 (*p* = 0.016). Regarding tumoral cells, statistically significant results were also obtained in the case of M2 at 48 h (*p* = 0.023) and M3 at 48 h (*p* = 0.038).

**Figure 3:**
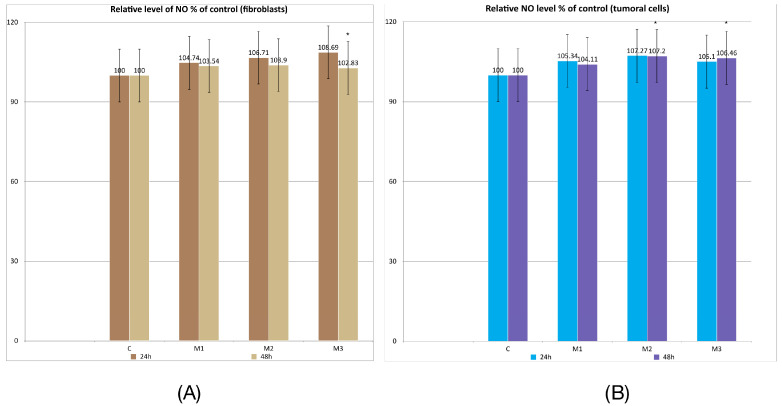
Griess test results. (**A**) Relative level of NO % of control at 24 h and 48 h in fibroblasts; (**B**) relative level of NO % of control at 24 h and 48 h in tumoral cells. Results are means ± SD (*n* = 6) * *p* < 0.05.

These data may indicate a slight proinflammatory effect of these two types of materials following 24 h and 48 h incubation both on fibroblasts and tumoral cells (Figure 3A,B).

### 3.2. Fluorescent Staining Results

Mitochondrial activity is crucial in cells exposed to various materials, including dental materials, because mitochondria are the main subcellular targets of the toxicant and key regulators of the stress response. They not only produce the energy needed (ATP) to manage the impact of the materials but also undergo structural and functional changes that determine cell fate (survival or death) [36].

**Figure 4:**
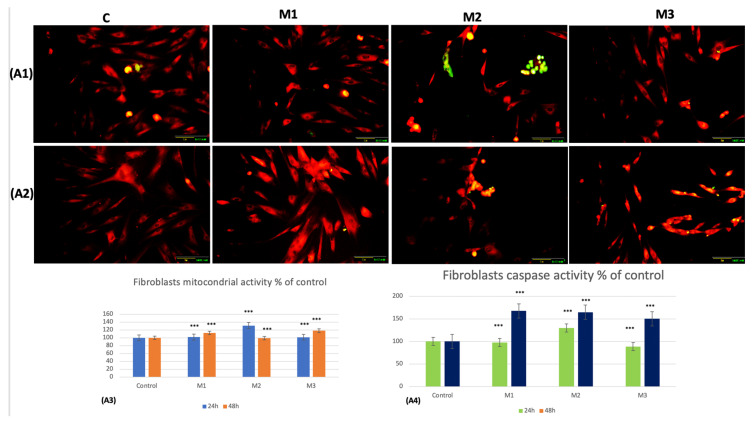

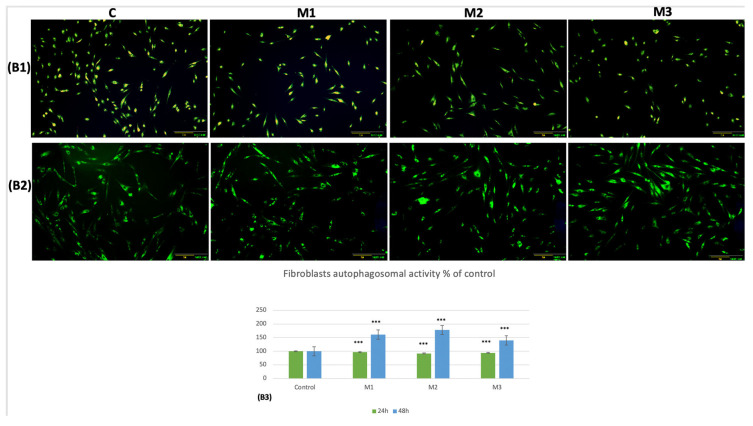
(**A1**,**A2**) Fluorescence detection of mitochondria (red) and PMMA-induced caspase-3/7 expression (green) at 24 h and 48 h; (**A3**,**A4**) statistical interpretation for mitochondria and caspase-3/7 expression in fibroblasts at 24 h and 48 h; (**B1**,**B2**) fluorescence detection and quantification of PMMA-induced autophagosome formation (green—autophagosomes) in fibroblasts at 24 h and 48 h; (**B3**) statistical interpretation for autophagosome formation at 24 h and 48 h. Scales: 20X objective and 100 μm. Results are means ± SD (*n* = 6) *** *p* < 0.001.

The results obtained for gingival fibroblasts indicated a statistically significant increase (*p* < 0.001) at both 24 h and 48 h. The highest increases in mitochondrial activity were recorded at 24 h in the case of M2 (31.4% *p* < 0.001) and at 48 h in M1 (12.6% *p* < 0.001) and M3 (18.3% *p* < 0.001) (Figure 4(A1–A4)).

**Figure 5:**
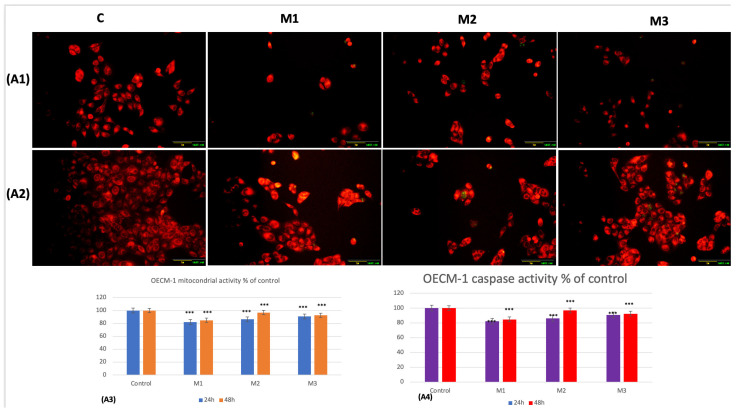

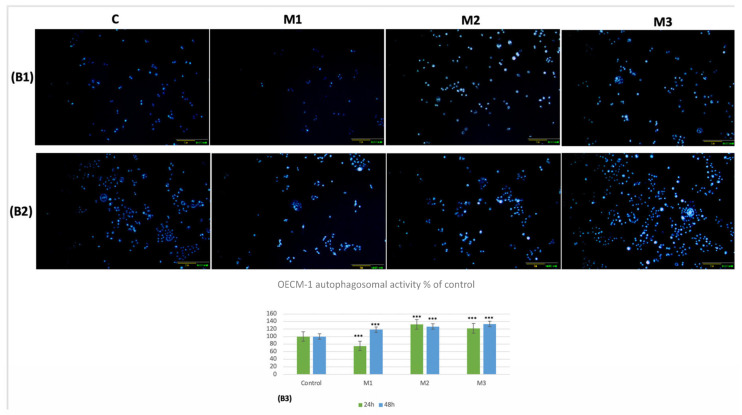
(**A1**,**A2**) Fluorescence detection of mitochondria (red) and PMMA-induced caspase-3/7 expression (green) at 24 h and 48 h in OECM-1; (**A3**,**A4**) statistical interpretation for mitochondria and caspase-3/7 expression in OECM-1 at 24 h and 48 h; (**B1**,**B2**) fluorescence detection and quantification of PMMA-induced autophagosome formation (blue—autophagosomes) in OECM-1 at 24 h and 48 h; (**B3**) statistical interpretation for autophagosome formation at 24 h and 48 h. Scales: 20x objective and 100 μm. Results are means ± SD (*n* = 6) *** *p* < 0.001.

However, in tumoral cells, statistically significant decreases (*p* < 0.001) in mitochondrial activity were recorded for the three materials included in the study. The most pronounced decrease was obtained for the 3D-printed material, M1, which was 17.8% (*p* < 0.001) after 24 h of incubation and 15.2% (*p* < 0.001) after 48 h of incubation (Figure 5(A1–A3)).

Regarding the formation of autophagosomes, it can be observed from both the images and the statistical analysis that fluorescence is reduced in gingival fibroblasts incubated for 24 h with the tested materials. The most pronounced decrease, 7.9% (*p* < 0.001), was recorded in M2, followed by a significant increase in all of the three materials included in the study at 48 h incubation (*p* < 0.001). After 48 h, autophagosome activity increased by 61.3% (*p* < 0.001) in M1, 78% (*p* < 0.001) in M2, and 39.8% (*p* < 0.001) in M3 (Figure 4(B1–B3)).

When testing tumoral cells, an increase in autophagosome formation was recorded at both 24 h and 48 h (*p* < 0.001) for the three materials, with one exception. At 24 h, M1 recorded a decrease in autophagosome formation by 25% (*p* < 0.001) followed by a sharp increase of 18.6% (*p* < 0.001) at 48 h, as shown by both the microscopy images and the statistical data (Figure 5(B1–B3)).

In addition, continuing the apoptosis evaluation, our results illustrated in Figure 4(A1–A4) and 5(A1,A2,A4) and Table 2 and Table 3 indicate a significant decrease in caspase-3/7 expression in fibroblasts at 24 h in M1 and M3 (*p* < 0.001) and a significant increased expression for 24 h in M2 (*p* < 0.001). At 48 h, the fibroblast experiment showed significant increased expression of caspase-3/7 expression in all the materials tested (*p* < 0.001).

Regarding tumoral cells, caspase-3/7 expression was decreased in all tested materials after 24 and 48 h of incubation, showing statistically significant values (*p* < 0.001).

**Table 2:** Variation in parameters tested % of control in gingival fibroblasts (* *p* < 0.05; ** *p* < 0.01; *** *p* < 0.001).

Test/Material	Control		Material 1		Material 2		Material 3	
	24 h	48 h	24 h	48 h	24 h	48 h	24 h	48 h
Cell viability	100	100	66.77 ***	52.45 ***	79.91 **	79.3 ***	85.92 ***	85.63 **
LDH	100	100	103.58	118.62	100	99.4 *	104.63	92.11
NO	100	100	104.74	103.54	106.71	103.9	108.69	102.83 *
Mitochondria	100	100	101.8 ***	112.6 ***	131.4 ***	99.2 ***	101.6 ***	118.3 ***
Caspase-3/7 activity	100	100	97.87 ***	167.818 ***	130.149 ***	164.936 ***	88.83 ***	150.167 ***
Autophagosome	100	100	96.6 ***	161.3 ***	92.1 ***	178 ***	94.2 ***	139.8 ***

**Table 3:** Variation in parameters tested % of control in tumoral OECM-1 cells (* *p* < 0.05; ** *p* < 0.01; *** *p* < 0.001).

Test/Material	Control		Material 1		Material 2		Material 3	
	24 h	48 h	24 h	48 h	24 h	48 h	24 h	48 h
Cell viability	100	100	69.04 ***	72.93 **	63.75 ***	64.91	104.76	61.79 ***
LDH	100	100	107.38	101.14	114.58	107.79	97.72	99.42
NO	100	100	105.34	104.11	107.27	107.2 *	105.1	106.46 *
Mitochondria	100	100	82.2 ***	84.8 ***	86.3 ***	96.9 ***	90.8 ***	92.5 ***
Caspase-3/7 activity	100	100	82.22 ***	84.77 ***	86.26 ***	96.85 ***	90.84 ***	92.45 ***
Autophagosome	100	100	75 ***	118.6 ***	132.3 ***	126.5 ***	121.8 ***	133.4 ***

## 4. Discussion

This study assessed in vitro biocompatibility and cellular stress responses to three dental materials (M1, M2, M3) using primary human gingival fibroblasts and the OECM-1 oral squamous carcinoma cell line. All materials affected metabolic activity/viability, mitochondrial function, autophagy, and apoptosis-related signaling, with the extent and direction of these effects varying by material, cell type, and time point. These differences highlight distinct cell-specific toxicity profiles and stress response behaviors, which are important for understanding clinical outcomes.

Our findings showed that all tested materials decreased fibroblast metabolic activity and viability compared to the control, with M1 causing the greatest reduction (about 33%—*p* < 0.001 at 24 h and about 48%—*p* < 0.001 at 48 h) probably due to a significant number of residual monomers [24,25,26,27,28]. M2 led to a moderate loss of fibroblast viability (20.09%—*p* < 0.01 at 24 h and 20.7%—*p* < 0.001 at 48%). Also, the results showed a moderate loss of cell viability in M3 (~14% *p* < 0.001 at 24 h and *p* = 0.013 at 48 h). In OECM-1 cells, M2 and M1 significantly reduced viability (~31–37%—*p* < 0.001), while M3 had no effect at 24 h but caused a notable decrease by 48 h (*p* < 0.001). These results suggest that M1 is the most cytotoxic to fibroblasts, M2 is relatively more cytotoxic to tumor cells early on, and M3 results in delayed cytotoxic effects in tumor cells. Additionally, LDH release changes were mostly minor, with a statistically significant decrease observed only for M2 on fibroblasts at 48 h. LDH is a stable cytoplasmic enzyme present in almost all cell types. Under normal conditions, it remains inside the cell. When the cell membrane is damaged, LDH leaks into the surrounding culture medium [45,46]. LDH release directly reflects loss of membrane integrity, which occurs during necrosis, chemical toxicity, or late-stage apoptosis. Higher LDH levels might reflect cell membrane damage caused by the dental material. If a dental material causes significant LDH release in vitro, it may lead to pulp inflammation, tissue necrosis, post-operatory sensitivity, or delayed healing [45,47,48]. The slight increase in LDH levels, despite notable decreases in metabolic viability, indicates that at least part of the viability loss may result from metabolic or mitochondrial dysfunction or programmed cell death, rather than from immediate necrotic membrane rupture. In a recent study, Hakan K. evaluated cell viability and cytotoxicity by measuring, among other parameters, LDH, and the results showed that some of the dental materials led to an increased level of LDH [48]. In another recent study, Wezgoviec et al. evaluated the cytotoxicity of 3D-printed dental materials and concluded that, although cell viability and LDH levels were altered, they did not reach alarming levels to be considered non-biocompatible [49].

Regarding the Griess test results, NO levels increased modestly (approximately 3–9%), with several statistically significant rises—particularly M2 and M3 in tumor cells (*p* = 0.023 in M2 and *p* = 0.038 in M3) and M3 in fibroblasts at certain times—indicating a mild proinflammatory or nitrosative stress response (*p* = 0.016). NO is a reactive free radical produced by cells via nitric oxide synthase (NOS) enzymes. In vitro, NO production is commonly measured indirectly by detecting nitrite (NO_2_^−^) accumulation using the Griess reaction [50]. NO plays dual roles as a physiological signaling molecule and as a mediator of inflammation and oxidative stress. Dental materials may release residual monomers, formaldehyde, or acidic by-products. These substances can stimulate or suppress NO production in oral cells. Gingival fibroblasts are primary cells involved in periodontal tissue maintenance and wound healing. Increased NO production may suggest activation of inducible nitric oxide synthase (iNOS), inflammatory stimulation, or cellular stress response [51,52]. Excess NO may lead to oxidative damage, delayed healing, and impaired collagen synthesis. NO can react with superoxide (O_2_^−^) to form peroxynitrite (ONOO^−^), a highly reactive species that damages lipids, alters proteins, and induces DNA damage [52]. Thus, elevated NO levels may indicate that a dental material induces oxidative toxicity [52,53]. In tumor cells, NO has a complex role since it is able to have either an anti-tumor or pro-tumor effect. High concentrations of NO may induce apoptosis, cause DNA damage, and inhibit tumor cell proliferation. Some dental materials may reduce tumor cell viability via oxidative stress mechanisms involving NO. Chronic, low-level NO production can promote angiogenesis, support tumor progression, and enhance cell migration. Thus, the NO test helps determine whether dental materials stimulate tumor growth pathways or induce anti-tumoral cytotoxicity [54,55].

The small magnitude suggests adaptive or sublethal inflammatory signaling rather than strong inflammatory cytotoxicity.

Moreover, our results showed a statistically significant increase in fibroblast mitochondrial activity, notably for M2 at 24 h (*p* < 0.001) and M1/M3 at 48 h (*p* < 0.001). OECM-1 cells exhibited significant mitochondrial suppression for all materials (most pronounced for M1, *p* < 0.001). Increased mitochondrial activity in fibroblasts could reflect compensatory upregulation of metabolism/biogenesis in response to stress, whereas decreased mitochondrial function in tumor cells is consistent with mitochondrial damage, leading to reduced ATP production and contributing to loss of viability. Another explanation could be that, given that the fluorescence-based assay used to assess mitochondrial activity cannot reliably distinguish whether mitochondria yield a positive signal are viable or dysfunctional, the observed decrease in cellular viability is not necessarily correlated with the fluorescence-determined mitochondrial activity. This is because the mitochondrial membrane may not have lost its fluorescence at the time of testing, as depolarization may not yet have occurred. Following our searches, we did not find similar studies that address mitochondrial activity in the context of the biocompatibility of PMMA-based dental materials, either on human gingival fibroblasts or on oral tumor cells.

Furthermore, our data revealed that autophagosome formation increased robustly by 48 h in fibroblasts for all materials (large fold increases, *p* < 0.001) but showed a mixed pattern in tumor cells (initial decrease with M1 at 24 h, *p* < 0.001, then increases by 48 h, *p* < 0.001; M2/M3 generally increased autophagy *p* < 0.001). Autophagy is a cellular self-cleaning process that maintains cellular homeostasis, prevents accumulation of toxic components, and is triggered by oxidative stress, nutrient deprivation, and cytotoxic compounds. Studies showed that residual monomers induce ROS, which will lead to mild stress and trigger autophagy as a protective response [56]. Recent studies showed that autophagosome formation in oral carcinoma cells due to PMMA nanocomposites protects cells under moderate stress [57,58]. However, an excess of monomers can be overwhelming for autophagy, and then cells head towards apoptosis, as another study shows [59].

Also, caspase-3/7 expression increased significantly in fibroblasts at 48 h for all materials (*p* < 0.001), indicating activation of the executioner caspases and apoptotic progression. In contrast, caspase-3/7 activity in tumor cells decreased at 24 h (and remained lower, *p* < 0.001), despite increases in autophagy [60,61,62]. Caspase-3 and caspase-7 are critical executioners of apoptosis. Their activation leads to DNA fragmentation, chromatin condensation, membrane blebbing, cell shrinkage, and detachment [60,61,62]. Residual monomers or PMMA extracts can induce cellular stress. When stress is too high, cells trigger apoptosis: mitochondrial outer membrane permeabilization, caspase-3/7 activation, and cell dismantling. Caspase-3/7 activity assays are often used to measure the cytotoxicity of dental materials. High caspase-3/7 activity leads to apoptosis and means low biocompatibility, while moderate or no activation indicates that cells are surviving (sometimes with autophagy) [62,63]. Autophagy and caspase-3/7-mediated apoptosis are interconnected. Protective autophagy can delay caspase activation by removing damaged mitochondria but if autophagy is overwhelmed, it leads to the activation of caspase-3/7 and apoptosis proceeds [62,63]. Studies showed that in PMMA fibroblasts exposed to low residual monomer, autophagy predominates and caspase-3/7 is at a low level, while in high residual monomers, the level of caspase-3/7 is high and apoptosis dominates [62,63].

Together, these data suggest that in fibroblasts, the dominant response evolves towards apoptosis (caspase activation) with concurrent induction of autophagy (possibly as an early protective response that later fails), whereas in tumor cells, autophagy is more prominent and caspase-dependent apoptosis is suppressed or delayed—implying that differential cell death pathways are engaged. A recent study conducted on oral keratinocytes and oral fibroblasts kept in contact with 3D-printed oral devices showed that there are no significant changes in caspases 3/7 that would affect the biocompatibility of the materials [64].

Material-specific effects: M1 (3D-printed material) caused the greatest reduction in fibroblast viability and the most significant mitochondrial suppression in tumor cells, indicating possible leachable substances or surface characteristics that strongly disrupt cell metabolism. M2 triggered earlier cytotoxic and LDH responses in tumor cells, suggesting distinct chemical or physical interactions. M3 exhibited the least fibroblast toxicity but a delayed tumor cytotoxic effect, pointing to time-dependent release or cumulative impacts. The combined pattern of mitochondrial dysfunction, increased autophagy, and eventual caspase activation in fibroblasts is consistent with a classical stress-to-apoptosis pathway: initial mitochondrial perturbation triggers mitophagy/autophagy as an adaptive response, but persistent damage leads to mitochondrial outer membrane permeabilization and caspase-mediated apoptosis. The modest LDH release supports apoptosis rather than necrosis as the predominant death mode in fibroblasts. However, in tumor cells, mitochondrial suppression with concurrent autophagy upregulation and reduced caspase activity may indicate cytoprotective autophagy or a non-apoptotic cell death route (e.g., autophagic cell death, necroptosis, or metabolic catastrophe). The early sensitivity of OECM-1 to M2 (viability decline, *p* < 0.001, and LDH increase at 24 h) suggests that M2 may induce faster membrane perturbation or a more lytic mechanism in tumor cells relative to fibroblasts.

The pattern of mitochondrial impairment and activation of autophagy/apoptosis induced by dental materials aligns with the literature showing that many resin-based and polymeric dental materials can impair mitochondrial function, generate oxidative/nitrosative stress, and trigger autophagy/apoptosis in oral cells. The cell-type specificity observed here (greater apoptotic signaling in primary fibroblasts versus autophagy-dominant responses in tumor cells) is consistent with known differences in stress responses between non-transformed and cancer cells (tumor cells often rely on autophagy for survival under metabolic stress and may resist caspase-mediated apoptosis). The modest NO increases align with reports that some dental materials induce mild inflammatory mediator release from oral cells, but the small magnitudes here suggest limited pro-inflammatory potential under the tested conditions.

The present study is subject to several limitations that should be considered when interpreting the findings. First, the relatively small sample size (*n* = 6) limits the statistical power and generalizability of the results; therefore, the observed effects should be regarded as preliminary and warrant confirmation in studies with larger cohorts. Second, only a single specimen dimension was evaluated, and variations in thickness or diameter may influence cellular responses, potentially leading to different biological outcomes. Finally, the absence of physicochemical characterization of the tested materials, particularly with respect to residual monomer content, represents an additional limitation; in this regard, the interpretation of results relied on previously published data from the scientific literature. Moreover, the use of in vitro monoculture assays constitutes an inherent limitation, as such models cannot fully replicate the complexity of oral tissues, including the interactions among multiple cell types, extracellular matrix components, immune responses, and fluid dynamics. Consequently, the findings may not accurately predict in vivo behavior, particularly with respect to long-term outcomes. Given the variability and sometimes conflicting data reported in the literature, the present study also aimed to explore underlying biological mechanisms in greater depth by comparatively evaluating apoptosis and autophagy in relation to cellular viability and cytotoxicity, in order to better understand the effects of these dental materials on both normal and tumor-derived oral cells.

## 5. Conclusions

The present study evaluated the in vitro biocompatibility of three PMMA-based dental materials manufactured using different fabrication techniques—3D printing (FotoDent Guide—M1), CAD/CAM milling (Aidite Temp—M2), and self-polymerization (Duracryl—M3)—on both normal gingival fibroblasts (HFIB-G) and human oral squamous carcinoma cells (OECM-1). Biological assessments were performed according to ISO 10993-5:2009 (viability cut-off 70%), allowing the evaluation of cytotoxicity and cellular stress responses relevant to clinical oral environments.

The results of the MTT assay indicated that all materials produced a reduction in fibroblast viability compared with control cells, with the most pronounced decrease observed for the 3D-printed material M1, while the self-polymerized material M3 showed the highest level of cellular tolerance. In tumoral OECM-1 cells, all materials also reduced cell viability, particularly M2 and M3 after 48 h incubation, suggesting a differential cellular response depending on the biological model.

Cytotoxicity evaluation through the LDH assay revealed generally low membrane damage. Nitric oxide quantification showed only moderate increases in NO production, indicating a mild inflammatory response.

Fluorescence analysis further highlighted cellular stress responses. In gingival fibroblasts, mitochondrial activity increased significantly following exposure to the tested materials, suggesting an adaptive metabolic response to material-induced stress. In contrast, tumoral OECM-1 cells exhibited significant reductions in mitochondrial activity, especially in M1. Autophagy analysis demonstrated an initial decrease in fibroblasts at 24 h followed by a marked increase after 48 h, indicating activation of cellular survival mechanisms. In tumoral cells, autophagosome formation increased in most experimental conditions, suggesting a stress-induced adaptive response. Additionally, apoptosis evaluation revealed increased caspase-3/7 expression in fibroblasts after 48 h exposure to all materials, while in tumoral cells, caspase activity decreased after 24 h incubation.

Overall, the findings indicate that all three PMMA-based materials exhibit more than 70% biocompatibility profiles according to ISO 10993-5:2009, although the magnitude and type of cellular response vary depending on the manufacturing technique and the cellular model used. Among the tested materials, the self-polymerized resin (M3) demonstrated the best compatibility with gingival fibroblasts, whereas the 3D-printed and CAD/CAM milled materials showed more pronounced effects on cell viability and metabolic activity.

Importantly, the use of both normal gingival fibroblasts and oral squamous carcinoma cells provides valuable insight into the biological behavior of these materials in both physiological and pathological conditions, which is particularly relevant for prosthetic rehabilitation in patients with a history of oral cancer. Nevertheless, further long-term in vitro studies and in vivo investigations are required to better understand the complex interactions between PMMA-based materials and oral tissues.

## Data Availability

The original contributions presented in this study are included in the article. Further inquiries can be directed to the corresponding authors.
